# Exploration of effective electroencephalography features for the recognition of different valence emotions

**DOI:** 10.3389/fnins.2022.1010951

**Published:** 2022-10-17

**Authors:** Kai Yang, Li Tong, Ying Zeng, Runnan Lu, Rongkai Zhang, Yuanlong Gao, Bin Yan

**Affiliations:** Henan Key Laboratory of Imaging and Intelligent Processing, People’s Liberation Army (PLA), Strategy Support Force Information Engineering University, Zhengzhou, China

**Keywords:** emotion recognition, emotion valence, EEG, feature extraction, feature selection

## Abstract

Recent studies have shown that the recognition and monitoring of different valence emotions can effectively avoid the occurrence of human errors due to the decline in cognitive ability. The quality of features directly affects emotion recognition results, so this manuscript explores the effective electroencephalography (EEG) features for the recognition of different valence emotions. First, 110 EEG features were extracted from the time domain, frequency domain, time-frequency domain, spatial domain, and brain network, including all the current mainly used features. Then, the classification performance, computing time, and important electrodes of each feature were systematically compared and analyzed on the self-built dataset involving 40 subjects and the public dataset DEAP. The experimental results show that the first-order difference, second-order difference, high-frequency power, and high-frequency differential entropy features perform better in the recognition of different valence emotions. Also, the time-domain features, especially the first-order difference features and second-order difference features, have less computing time, so they are suitable for real-time emotion recognition applications. Besides, the features extracted from the frontal, temporal, and occipital lobes are more effective than others for the recognition of different valence emotions. Especially, when the number of electrodes is reduced by 3/4, the classification accuracy of using features from 16 electrodes located in these brain regions is 91.8%, which is only about 2% lower than that of using all electrodes. The study results can provide an important reference for feature extraction and selection in emotion recognition based on EEG.

## Introduction

Emotions play an important role in our daily life because they can affect people’s work efficiency, decision-making, memory et al. Compared with neutral emotions, positive and negative emotions tend to decline our cognitive ability (Qian et al., 2015; Wang and Liu, 2020). If people’s cognitive abilities for special jobs are affected by their emotions, there may be serious consequences. Therefore, if the emotions that lead to cognitive decline can be effectively identified and timely warned, most of the adverse consequences caused by cognitive decline can be avoided.

Almost everyone experienced the change from a positive emotional state to a negative emotional state at some point. According to the continuous emotion model proposed by Lang et al. (2001), the change of positive emotion and negative emotion indicates the variations of emotion valence. Previous studies have pointed out that different valence emotions have different effects on cognition performance. For example, although an extremely negative emotional state may reduce cognitive abilities significantly, a moderately negative emotional state can enhance alertness and responsiveness (Chen et al., 2013). Besides, people’s memory and judgment abilities tend to be weakened in negative valence emotional states, which makes it easier to make irrational decisions. Compared with negative emotional states, most positive emotional states are usually harmless and may even improve people’s cognitive abilities. Blair et al. (2007) investigated the interaction between positive, neutral, and negative valence emotions and goal-directed processing tasks. They found that positive and negative valence stimuli have a greater impact on goal-directed tasks than neutral valence stimuli. Based on event-related potentials (ERP) and functional magnetic resonance imaging (fMRI) research, Li et al. (2006) found that negative valence emotion has a greater impact on spatial working memory than on verbal working memory. Yuan et al. (2007) explored people’s sensitivity to valence differences in emotional stimuli by using different valence pictures with no significant difference in arousal as stimulus materials, and they found that people are more sensitive to negative valence pictures. Meng et al. explored the influence of attention on human sensitivity to valence differences in emotional stimuli. They found that the ERP amplitude of extremely negative valence pictures was greater than that of moderately negative and neutral pictures within 150–250, 250–350, and 350–450 ms after the pictures were presented (Meng et al., 2009). As described above, people have different sensitivities to emotions with different valence, and different valence emotions have different effects on people’s cognitive performance. However, it is not enough to claim that emotions with different valence have an impact on people’s cognition. When people are in these emotional states, if early warning can be given to ask them to stop working, serious consequences may be avoided. The premise of early warning is to accurately classify different valence emotions.

Electroencephalography has the advantages of non-invasiveness, high time resolution, and good portability, and it has been widely used in emotion recognition research (Hu et al., 2019). Feature extraction is a key step in emotion recognition based on EEG. The quality of features will directly affect the accuracy of emotion recognition (Wang and Wang, 2021). EEG features in emotion recognition can be mainly divided into time domain (statistical features), frequency domain, time-frequency domain, spatial asymmetry, and brain network features (Li et al., 2016; Gonuguntla et al., 2020). Time-domain features mainly include first-order difference, second-order difference, fractal dimension, sample entropy, approximate entropy, and standard deviation (Lan et al., 2016). Frequency-domain features mainly include power spectral density and power (An et al., 2021). Time-frequency domain features are mainly features extracted based on the discrete Fourier transform or Hilbert Huang transform (Khare and Bajaj, 2021). Differential entropy is the most used time-frequency feature, which has achieved the highest classification accuracy in multiple studies (Nie et al., 2011; Zheng et al., 2019). Spatial asymmetry feature refers to the difference or ratio of features from left and right hemisphere electrodes (Zheng and Lu, 2015). Additionally, the brain network feature including the connection between electrodes is a new feature in recent years, which is increasingly used in emotion recognition and has achieved good results. Li et al. (2019) proposed to fuse local features extracted from a single electrode with brain network features containing global information, which improved the performance of emotion classification. Our previous study also compared the performance of EEG network features of different frequency bands, and the results showed that the high gamma band brain network features were more closely related to emotion (Yang et al., 2020). Wu et al. (2022) pointed out that the brain network features representing the relationship between different electrodes have better classification performance than the differential entropy extracted from a single electrode. Though there are currently many types of features used in emotion recognition, there is no agreement upon which features are most appropriate. Since the computational complexity of multiple feature extraction is high, and the extraction of some features requires rich experience and professional knowledge, only a few studies compared the performance of different features. For example, Jenke et al. (2014) compared multiple features on a self-recorded dataset of 16 subjects and five emotions. Li et al. explored two set of features for cross-subject emotion recognition, and the Hjorth parameter of mobility in the beta rhythm achieved the best mean recognition. Moreover, using multiple electrodes will need more preparation time and lead to unfriendly user experience. Many studies have been conducted on the electrode selection in emotion recognition. For instance, Zheng and Lu (2015) explored the four most important electrodes on the SEED dataset, and they found FT7, FT8, T7, and T8 are most important electrodes. Li et al. (2018) explored the most important 10, 14, and 18 electrodes on the DEAP dataset, and most of these electrodes are distributed in the frontal brain region.

Previous studies have investigated feature extraction and selection in emotion recognition, but a major limitation of these studies is that they did not explore the effective features for the recognition of different valence emotions. Meanwhile, most studies rely on a different and usually small dataset. This work aims to systematically explore effective features for the recognition of different valence emotions. According to previous studies, arousal has a nonspecific effect on valence (Johnson, 1995; Carretié et al., 1997). Firstly, this study selected five types of valence pictures from the Chinese Affective Picture System (CAPS) (Bai et al., 2005), including extremely negative, moderately negative, neutral, moderately positive, and extremely positive, which have no significant difference in arousal. Then, 110 features commonly used in other papers are extracted from five feature domains of time domain, frequency domain, time-frequency domain, spatial asymmetry, and brain network. Finally, the classification performance, calculation time, and important few electrodes of the features are systematically compared and analyzed on the self-built big dataset of 40 subjects and the public dataset DEAP (Koelstra, 2012).

## Materials and methods

### Participants

This experiment includes 40 healthy subjects (20 women) of native college students, aged 18–28 years old (average age 22 years old), one of whom is double handed, and the rest are right-handed. All subjects had no mental illness, did not take drugs that affected their mental state, and all subjects with normal mental state tested by Baker depression scale (Jackson-Koku, 2016) and Baker anxiety scale (Wilson et al., 1999), and all subjects’ with normal vision or corrected normal vision. Before the experiment, each subject was informed of the content and the purpose of the experiment and signed the informed consent. After the experiment, subjects received a certain amount experimental fee.

### Experiment procedure

Emotion pictures are divided into five categories according to valence: extremely negative (EN), moderately negative (MN), neutral, moderately positive (MP), and extremely positive (EP), and each category contains 30 pictures. The mean value and standard deviation of valence degrees of the different categories pictures are EN = 1.87/0.35, MN = 3.56/0.54, neutral = 5.6/0.49, MP = 6.28/0.17, and EP = 6.81/0.16, and the arousal degrees are EN = 5.54/0.16, MN = 5.5/0.2, neutral = 5.54/0.28, MP = 5.49/0.19, and EP = 5.57/0.18. There are significant differences between the valence degrees of the five categories of pictures (*P* < 0.01), and there was no significant difference (*P* > 0.05) between the arousal degrees.

The numbers in the digital picture are 2, 3, 4, 5, 6, 7, 8, and the number of numbers varies from 3 to 6. The content of the digital picture is 3*3, and numbers or “*” appear randomly at 9 positions. The left and right numbers are not adjacent in the picture, e.g., the left and right numbers will not be “2” and “3.” There are two types of digital pictures: consistent and inconsistent. The consistent situation is that there are more numbers with large values or fewer numbers with small values, while the inconsistent situation is that there are more numbers with small values or fewer numbers with large values.

Each trial begins with a white “+” for 2–4 s in the center of the screen with black background, then presents an emotion picture for 2000 ms, then presents a digital picture for 1000 ms, and then presents the valence and arousal rating pictures. When the subjects see the digital picture, they need to press the key quickly and accurately to determine which side of the numbers on the left and right is larger. If the number on the left is larger, the subjects should press the alphabet “Q” on the keyboard with the left index finger; if the number on the right is larger, they should press the number “0” on the keyboard with the right index finger, and the key response should be made within 1000 ms. The digital picture will disappear once the subjects press the keyboard, and the valence rating picture will be represented. If the key response is not made after 1000 ms, the valence rating picture will also be represented. Valence and arousal ratings are achieved by pressing keys 1–9 on the keyboard (Morris, 1995). The subjects take a 2-min break between blocks to eliminate the emotional impact of the previous block on the next block and alleviate the subjects’ mental fatigue. Five blocks are presented randomly, and 30 trials in each block are presented in random order. The experimental paradigm was conducted by Tobii Pro Lab software, and the subjects’ key response values and time were recorded.

### Data acquisition and preprocessing

This experiment was carried out in a professional laboratory with electromagnetic shielding condition and suitable temperature and light. In the experiment, the subjects sat on a chair with adjustable height facing the screen, and their eyes were about 65 cm away from the screen. EEG signals were recoded with 64 channel G.HIamp system. During the experiment, the impedance of each electrode was kept below 10 KΩ, the electrodes were located according to the international 10–20 standard system. Electrode AFz was used as the ground electrode, electrode Fz and right earlobe were used as references, and the number of effective electrodes was 62. The EEG data sampling rate is 600 Hz. Online 0.1–100 Hz band-pass filtering and 48–52 Hz notch filtering are conducted during EEG data acquisition. Meanwhile, eye gaze data is collected by the Tobii Nano device with a sampling rate of 60 Hz. All these multi-modal data compose the Emotion-Stroop dataset (ESD). This paper only uses EEG data. In the pre-processing procedure, the collected original EEG signals are segmented first, and the data of 500 ms before picture presentation and the data of 2000 ms during picture presentation are extracted. The subsequent preprocessing mainly includes 0.1–80 Hz filtering and performing the blind-source analysis algorithm Fast-ICA (HyvRinen, 1999) to remove electrooculography (EOG) artifacts, average reference, and baseline correction.

### Feature extraction

This manuscript summarizes the commonly used EEG features and extraction methods in recent years. Jenke et al. have extensively studied feature extraction (Jenke et al., 2014), and our study supplements some recently developed important features on their basis. The features are roughly divided into five feature domains: time domain, frequency domain, time-frequency domain, spatial asymmetry, and brain network features. In this paper, the total number of EEG electrodes is denoted as ch, the number of time points per electrode is denoted as N, and the EEG data of a certain electrode at a certain time is denoted as x (n). The specific extraction methods of each feature are as follows.

#### Time-domain features

The time-domain features extracted in this paper include standard deviation, first-order difference, second-order difference, normalized first-order difference, normalized second-order difference, fractal dimension, sample entropy, and approximate entropy. The specific calculation methods are as follows.

• Standard deviation (Std)

(1) $$\delta_{c\,h}=\sqrt{\frac{1}{N}\,\sum_{n=1}^{N}{\left(x\,\left(n\right)-\frac{1}{N}\,\sum_{n=1}^{N}x\,\left(n\right)\right)}^{2}}$$

• First-order difference (Fir-dif)

(2) $$f\,i\,r\,s\,t\,\_\,d\,i\,f\,f_{c\,h}=\frac{1}{N-1}\,\sum_{n=1}^{N-1}\left|x\,\left(n+1\right)-x\,\left(n\right)\right|$$

• Normalized first-order difference (N-fir-dif)

(3) $$N\,o\,r\,\_\,f\,i\,r\,s\,t\,\_\,d\,i\,f\,f_{c\,h}=\frac{f\,i\,r\,s\,t\,\_\,d\,i\,f\,f_{c\,h}}{\delta_{c\,h}}$$

• Second-order difference (Sec-dif)

(4) $$\sec o\,n\,d\,\_\,d\,i\,f\,f_{c\,h}=\frac{1}{N-2}\,\sum_{n=1}^{N-2}\left|x\,\left(n+2\right)-x\,\left(n\right)\right|$$

• Normalized second-order difference (N-sec-dif)

(5) $$N\,o\,r\,\_\,\sec o\,n\,d\,\_\,d\,i\,f\,f_{c\,h}=\frac{\sec o\,n\,d\,\_\,d\,i\,f\,f_{c\,h}}{\delta_{c\,h}}$$

• Fractal Dimension (FD)

Fractal dimension (FD) is a non-linear feature used to measure the complexity of EEG signals. The commonly used calculation methods of fractal dimension are box dimension fractal and Higuchi fractal. In this manuscript, the Higuchi fractal is used to calculate the fractal dimension (Lan et al., 2016), and the specific calculation process is as follows:

Let the initial sequential EEG signal be _*X*(1)_, _*X*(2)_, …,_*X(N)*_. The EEG signal sequence is sampled at every _*k*_ points as follows:


(6) $$\begin{array}{c} X_{k}^{m}:X\,\left(m\right),X\,\left(m+k\right),\ldots ,X\,\left(m+\left[\frac{N-m}{k}\right]\cdot k\right)\\ m=1,2,3,\ldots ,k \end{array}$$

where *m* is the initial time of sampling, and *k* is the time interval of sampling.

Define *m* sampling points as *L*_*k*_(*m*):


(7) $$L_{k}\left(m\right)=\frac{1}{k}\cdot$$

$$\left[\frac{\left(\sum_{i=1}^{\left\lfloor \frac{N-m}{k}\right\rfloor }\left|X\,\left(m+i\,k\right)-X\,\left(m+\left(i-1\right)\right)\,k\right|\right)\,\left(N-1\right)}{\left\lfloor \frac{N-m}{k}\right\rfloor \,k}\right]$$

Denote the mean value of all the sampling points in *L*_*k*_(*m*) as *L*(*k*). FD is inversely proportional to *L*(*k*) as follows:


(8) $$F\,D=-\lim_{k\to \infty }\frac{\log\left\langle L\,\left(k\right)\right\rangle }{\log k}$$

• Approximate Entropy (ApEn)

Approximate entropy (ApEn) reflects the possibility of new information in time series. The larger the approximate entropy, the more complex the time series. Denote an integer as m and a real number as r. Then, an m-dimensional vector *x*(1),*x*(2), …*x*(N−m + 1) can be constructed from the original EEG signal, where *x*(i) = [X(1),X(2),…X(i + M−1))] counts the number of vectors that meet the following conditions:


(9) $$C_{i}^{m}\left(r\right)=\left(numberofX\left(j\right)such\;that\;d\left[X\left(i\right),X\left(j\right)\right]\leq r\right)/\left(N-m+1\right)$$

where *d*[*X*(*i*),*X*(*j*)] = *max*⁡|*X*(*i*)−*X*(*j*)|,


(10) $$\Phi^{m}\,\left(r\right)={\left(N-m+1\right)}^{-1}\,\sum_{i=1}^{N-m+1}l\,o\,g\,\left(C_{i}^{m}\left(r\right)\right)$$

Then, the approximate entropy ApEn is defined as:


(11) $$A\,p\,E\,n=\Phi^{m}\,\left(r\right)-\Phi^{m+1}\,\left(r\right)$$

• Sample Entropy (SamEn)

Sample entropy (SamEn) is improved based on approximate entropy by eliminating the problem of approximate entropy self-matching, which is equivalent to optimizing the approximate entropy. In the calculation *d*[*X*(*i*),*X*(*j*)] = *max*⁡|*X*(*i*)−*X*(*j*)|, *i*≠*j*


(12) $$C^{m}\,\left(r\right)={\left(N-m+1\right)}^{-1}\,\sum_{i=1}^{N-m+1}\left(C_{i}^{m}\left(r\right)\right)$$

Then, SamEn is defined as:


(13) $$S\,a\,m\,p\,E\,n\,\left(m,r\right)=\lim_{N\to \infty }\left[-\ln\frac{C^{m+1}\,\left(r\right)}{C^{m}\,\left(r\right)}\right]$$

when n is a finite number. SamEn can be further expressed as:


(14) $$S\,a\,m\,p\,E\,n\,\left(m,r,N\right)=\ln C^{m}\,\left(r\right)-\ln C^{m+1}\,\left(r\right)$$

#### Frequency domain features

• Power Spectral Density (PSD)

Power spectral density (PSD) is commonly used to measure the frequency-domain information features of EEG signals. In this manuscript, power spectral density uses the p-welch method to calculate the frequency band power spectral density:


(15) $$p\,s\,d=\sum_{f\,l}^{f\,u}P\,\left(f\right)/\left(f\,u-f\,l\right)$$

where *P*(*f*) is the power spectral density at the frequency; *fl* is and *f*u are the lowest and highest frequency of the band of interest, respectively.

• Power (P)

Band power is based on short-time Fourier transform (STFT),


(16) $${\text{STFT}}_{x,\gamma }\,\left(n,f\right)=\int_{-\infty }^{+\infty }x\,\left(\tau \right)\,\gamma^{*}\,\left(n-\tau \right)\,e^{-j\,2\,\pi \,f\,\tau }\,d\tau =\,\int_{-\infty }^{+\infty }x\,\left(\tau \right)\,\gamma_{n,f}^{*}\,e^{-j\,2\,\pi \,f\,\tau }$$

(17) $$p\,o\,w\,e\,r=\sum_{f\,l}^{f\,u}{\left|S\,\left(n,f\right)\right|}^{2}$$

The power in six frequency bands is calculated for ESD data, including delta (1–4 Hz), theta (4–8 Hz), alpha (8–13 Hz), beta (13–30 Hz), gamma (30–50 Hz), and high gamma (50–80 Hz). Meanwhile, the power in four frequency bands is calculated for the DEAP dataset, including theta (4–8 Hz), alpha (8–13 Hz), beta (13–30 Hz), and gamma (30–50 Hz).

#### Time frequency domain features

• Different Entropy (DE)

The differential entropy (DE) feature is the most used feature at present, and its calculation is based on STFT. It calculates the differential entropy in six frequency bands of delta (1–4 Hz), theta (4–8 Hz), alpha (8–13 Hz), beta (13–30 Hz), gamma (30–50 Hz), and high gamma (50–80 Hz) of the ESD datasets, and it calculates the differential entropy in four frequency bands of theta (4–8 Hz), alpha (8–13 Hz), beta (13–30 Hz), and gamma (30- 50 Hz) of the DEAP datasets:


(18) $$D\,E=\log\left(\sum_{f\,l}^{f\,u}{\left|S\,T\,F\,T\,\left(n,f\right)\right|}^{2}\right)$$

#### Brain network features

The brain network connection matrix takes electrodes as network nodes to calculate the relationship between the data between electrodes, and the network can be mainly divided into a directed network and an undirected network. Here, only three commonly used undirected networks are considered for calculation: Pearson correlation (Pea), coherence (Coh), and phase lock value (PLV). Then, the clustering coefficient (CC), characteristic path length (CPL), and local efficiency (Le) are calculated based on the network connection matrix, and the global efficiency (Ge) is characterized by four commonly used network attributes (Van Straaten and Stam, 2013).

• Pearson correlation coefficient (Pea)

(19) $$p\,e\,a\,r\,s\,o\,n=\frac{\sum_{i=1}^{N}\left(X\,\left(i\right)-\bar{X}\right)\,\left(Y\,\left(i\right)-\bar{Y}\right)}{\sqrt{\sum_{i=1}^{N}{\left(X\,\left(i\right)-\bar{X}\right)}^{2}}\,\sqrt{\sum_{i=1}^{N}{\left(Y\,\left(i\right)-\bar{Y}\right)}^{2}}}$$

where *X*(*i*), *Y*(*i*) indicating the EEG value of two electrodes at time i.

• Coherence (Coh)

(20) $$C_{X\,Y}\,\left(f\right)=\frac{{\left|P_{X\,Y}\,\left(f\right)\right|}^{2}}{P_{X\,X}\,\left(f\right)\,P_{Y\,Y}\,\left(f\right)}$$

• Phase lock value (PLV)

(21) $$P\,L\,V=\left|\frac{1}{c\,h}\,\sum_{i=0}^{N-1}e^{i\,\left(\Phi_{x}\left(i\,t\right)-\Phi_{y}\left(i\,t\right)\right)}\right|$$

Where *ch* is the total number of EEG electrodes, *t* is the sampling period, Φ*_x_*(*it*) and Φ*_y_*(*it*) is the instantaneous phase of two electrodes *x*(*t*) and *y*(*t*) at time point *i*.

The CC describes the tightness and clustering characteristic of nodes in the brain network; the CPL is used to measure the connectivity degree of the network, and it represents the average length of the shortest path between any two nodes in the network; the Le is used to measure the information interaction ability in the local network, and the Ge describes the information transmission efficiency of the whole brain network. The definitions of the four network properties are given below, where n represents the number of nodes; Θ represents a node set; *w*_*ij*_ indicates the network connection value between nodes *i* and *j*; *d*_*ij*_ represents the shortest path length between nodes *i* and *j*.


(22) $$C\,C={\frac{\sum_{j,h\in \Theta }\left(w_{i\,j}\,w_{i\,h}\,w_{j\,h}\right)}{\sum_{j\in \Theta }w_{i\,j}\,\left(\sum_{j\in \Theta }w_{i\,j}-1\right)}}^{1/3}$$

(23) $$C\,P\,L=\frac{1}{n}\,\sum_{i\in \Theta }\frac{\sum_{i\in \Theta ,j\neq i}d_{i\,j}}{n-1}$$

(24) $$L\,e={\frac{\sum_{j,h\in \Theta ,j\neq i}\left(w_{i\,j}\,w_{i\,h}\,{\left[d_{j\,h}\,\left(\Theta_{i}\right)\right]}^{-1}\right)}{\sum_{j\in \Theta }w_{i\,j}\,\left(\sum_{j\in \Theta }w_{i\,j}-1\right)}}^{1/3}$$

(25) $$G\,e=\frac{1}{n}\,\sum_{i\in \Theta }{\frac{\sum_{j\in \Theta ,j\neq i}\left(d_{i\,j}\right)}{n-1}}^{-1}$$

#### Spatial asymmetry features

Spatial asymmetry features are based on the asymmetry characteristic of the brain reported in previous studies, mainly including differential asymmetry (DA) and rational asymmetry (RA) (Duan et al., 2012). The DA features represent the subtraction values of the features from the left and right hemisphere electrodes, and the RA features represent the ratio of the features from the left and right hemisphere electrodes. DA and RA are defined as follows:

• Differential Asymmetry (DA)

(26) $$D\,A=f\,e\,a_{l}-f\,e\,a_{r}$$

• Rational Asymmetry (RA)

(27) $$R\,A=f\,e\,a_{l}/f\,e\,a_{r}$$

Where *fea*_*l*_ and *fea*_*r*_ represent the features extracted by the symmetrical position electrodes of the left and right hemispheres, respectively. The asymmetry features of ESD data include 27 pairs of electrodes, and the DEAP dataset includes 17 pairs of electrodes.

### Feature selection

The purpose of feature selection is to find the key electrodes for the recognition of different valence emotions and provide a foundation for using a few electrodes for emotion recognition in practical applications.

Currently, the commonly used feature selection methods mainly include Relief (Jia et al., 2013), min redundancy max relevance (mRMR) (Ding and Peng, 2005), and forward floating search (Bhadra and Bandyopadhyay, 2021). Among them, mRMR is the most famous feature selection algorithm and has been applied in many emotion recognition studies. mRMR exploits mutual information to characterize the performance of feature subsets. This study also used the mRMR feature selection algorithm to explore the key electrodes for emotion recognition. This study attempted to find the most important 1, 4, 8, and 16 electrodes for the recognition of different valence emotions. First, the most important 1, 4, 8, and 16 electrodes in each subject’s classification are determined based on mRMR. Then, the frequency of each electrode in 40 subjects is counted, the most selected electrodes are taken as key electrodes, and the electrodes’ location in the brain regions is also analyzed.

### Classification settings

Classification is to match features with emotions to obtain classification accuracy. Classifiers can be roughly divided into two categories. The first category is the current popular classifiers based on the deep neural network (DNN). These classifiers are mainly the convolution neural network (CNN) (Santamaria-Vazquez et al., 2020), recurrent neural network (RNN) (Lukosevicius and Jaeger, 2009), long and short-term memory (LSTM) (Peng et al., 2016), and graph revolutionary neural network (GCNN) (Zhong et al., 2020). In recent years, most of these DNN classifiers have achieved excellent classification results, and most of these DNN classifiers have re-extracted the input features (Chen et al., 2019). However, due to the “black box” characteristic of DNNs, the significance of re-extracted features cannot be clearly explained (Zhou et al., 2016). The other category is the traditional shallow classifiers, such as support vector machine (SVM) (Chen et al., 2020), k-nearest neighbor (KNN) (Keller et al., 2012), linear discriminate analysis (LDA) (Saeedreza et al., 2009), extremely learning machine (ELM) (Huang et al., 2006), random forest (RF) (Liaw and Wiener, 2002), naive Bayes (NB) (Rish, 2001), discriminant analysis classifier (DAC) (Alkan and Günay, 2012), and boosting (Sun et al., 2007). Usually, the hyper-parameters in the DNN need to be tuned (He et al., 2019), and this procedure will change input features to unknown features (Saha and Fels, 2019), which makes it difficult to objectively compare the performance of different features. Most of the shallow classifiers do not need to tune complex parameters. Therefore, this paper used the six commonly used shallow classifiers (SVM, KNN, RF, NB, DAC, and boosting) to compare the classification performance of different features. Meanwhile, LibSVM (Chen et al., 2020) was used with a linear kernel, and the parameter was set to “−s 0 −t 0.”

Two experiments were conducted on the classification of the ESD dataset. The first is a two-category experiment that separates EN valence emotions from other valence emotions. In this experiment, 30 samples of EN valence emotions of each subject are regarded as one category, and 120 samples of MN, MP, EP, and neutral emotions are mixed as another category. Because it is a mixture of 4 emotions, each subject is sampled 4 times, 5-fold cross-validation is used for classification, and the average accuracy of 4*5 times classification is taken as the final accuracy of each subject. The second experiment is to classify five types of valence emotions: EN, MN, MP, EP, and neutral. Five-fold cross-validation is used in the classification, and the average accuracy five times classification is used as the final classification accuracy of each subject.

The DEAP dataset contains multimodal data such as EEG, galvanic skin response and respiratory rate during 32 subjects watching 40 1-min music videos with different valence and arousal. The EEG signals are collected from 32 active electrodes arranged according to the 10–20 international system. Only EEG signals are used in this study. To determine the effective features of different valence emotion classification in the DEAP dataset, the samples with arousal ratings in the range of 3.5–6 of each subject are selected, and then these selected samples are divided into high valence emotion samples with valence ratings greater than 5 and low valence emotion samples with valence ratings less than 5. Since the two types of samples of some subjects are unbalanced, this study calculates the sample number of two categories and then randomly selects the same number of samples as the fewer samples category from the category with more samples. According to experimental experience and previous research, emotion does not occur immediately after the stimulus is presented, so only the last 30 s of data induced by each video in the DEAP dataset were used in the experiment, and the data were divided into 5 s by non-overlapping segmentation. The preprocessing method of the EEG data in the DEAP dataset is consistent with that in the original dataset.

## Results

### Behavior data

The mean reaction time (RT) of 40 subjects under five different valence emotions is presented in Table 1. Compared with neutral valence emotion, the other four valence emotions all cause the subjects to react more slowly. This indicates that different valence emotions can affect the subjects’ reaction ability. When subjects are under an extremely negative valence emotion, the reaction time is longer than that under other valence emotions (*p* < 0.05), and the reaction time is the shortest when the subjects are under natural emotions. Subjects respond to trials involving MP and EP more slowly than to natural trials, but it does not reach significance (*P* = 0.06). So, different valence emotions can affect cognitive performance.

**TABLE 1:** Mean reaction time under different valence emotions.

Emotion	RT (ms)	Significance
EN	617.8	**
MN	599.6	*
Natural	585.0	–
MP	596.4	*
EP	581.7	–

**P < 0.05; *, in the edge of significance; -, no significance.

### Classification performance of different features

The classification performance of each feature for different valence emotions was first compared on the ESD dataset and DEAP dataset. Then, the highest classification accuracy of the time domain, frequency domain, time-frequency domain, DA, RA, and brain network features by using the classifiers of KNN, RF, SVM, DAC, Bayes, and boosting were presented, respectively. Two-class and five-class classification experiments were conducted on the ESD dataset, and high and low valence two-class classification experiment was carried out on the DEAP dataset.

#### Classification performance on the ESD dataset

**FIGURE 1:**
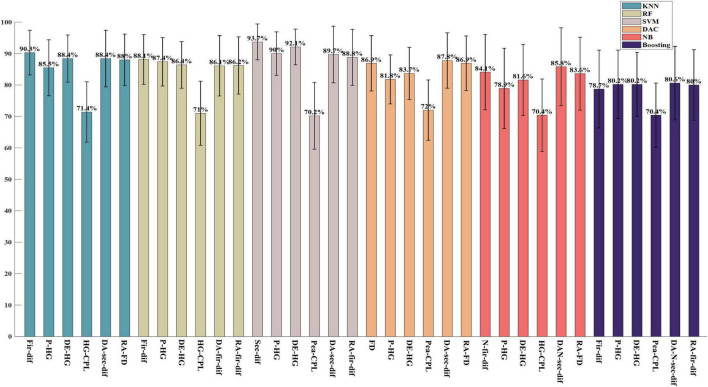
The highest two-class classification accuracy in each feature domain on the ESD. The block which including six bars of same color is the results of same classifier and from left to right, it is the classification results by classifiers KNN, RF, SVM, DAC, NB, and Boosting, respectively.

As shown in Figure 1, all features achieved a classification accuracy of no less than 70% in recognizing the EN emotion from other valence emotions, and the highest classification accuracy of 93.7% is achieved with the second-order difference by the SVM classifier. According to the classification results, time-domain features, first-order difference features, and second-order difference features perform better. The classification accuracy of first-order difference features under the KNN classifier is 90.3%, and the classification accuracy of second-order difference features under the SVM classifier is 93.7%. High gamma (HG) band power achieved the highest classification accuracy of 90% under the SVM classifier among frequency-domain features. The high gamma band DE obtained the highest accuracy in the time-frequency domain, and the classification accuracy under the SVM classifier is 92.1%. For brain network features, the CPL of the high gamma band coherence network performed the best and achieved a classification accuracy of 71.4% under KNN. For spatial asymmetric features, the DA feature extracted from second-order difference features obtained the highest classification accuracy of 89.7% under the SVM classifier, and the RA features extracted from first-order difference features performed the best and achieved a classification accuracy of 88.8% under the SVM classifier. The results show that the performance of first-order difference and second-order difference features in the time domain can achieve higher accuracy than that of power and differential entropy, which are mostly used in previous studies. In addition, it can be found that frequency-domain features, time-frequency domain features, and coherence network features in higher frequency bands performed better than those in lower frequency bands, and the high gamma band features achieved the highest classification accuracy. By comparing the performance of different classifiers, it can be found that the SVM classifier performed better in binary classification on ESD datasets.

**FIGURE 2:**
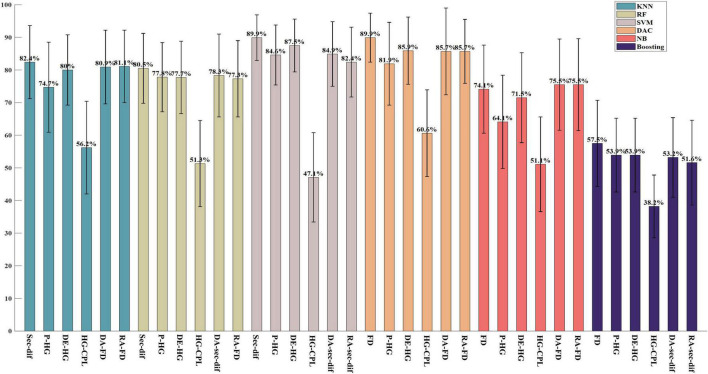
The highest five-class classification accuracy in each feature domain on the ESD. The block which including six bars of same color is the results of same classifier and from left to right, it is the classification results by classifiers KNN, RF, SVM, DAC, NB, and Boosting, respectively.

As shown in Figure 2, for the classification of five-class valence emotions on ESD datasets, the highest classification accuracy of 89.9% is achieved by second-order difference features under the SVM classifier and by FD features under the DAC classifier. Among time-domain features, second-order difference features and FD features achieved the best performance. In the frequency domain, high gamma band power performed best, and the classification accuracy achieved by the SVM classifier was 84.6%. Among time-frequency features, the high gamma band DE achieved the highest classification accuracy of 87.5% under the SVM classifier. The high gamma band CPL of the coherence network performed the best among all brain network properties, and the classification accuracy based on the DAC classifier is 60.6%. For spatial asymmetric features, both the DA features and RA features extracted from FD features achieved the highest classification accuracy of 85.7% under the DAC classifier. When classifying five kinds of valence emotions, it was also found that the accuracy of features in higher frequency bands is higher than that in lower frequency bands, and the high gamma band feature obtained the highest classification accuracy. Comparing the performance of different classifiers, it can be found that SVM and DAC classifiers have better classification performance in the classification of five-class valence emotions on ESD datasets.

#### Classification performance on the DEAP dataset

**FIGURE 3:**
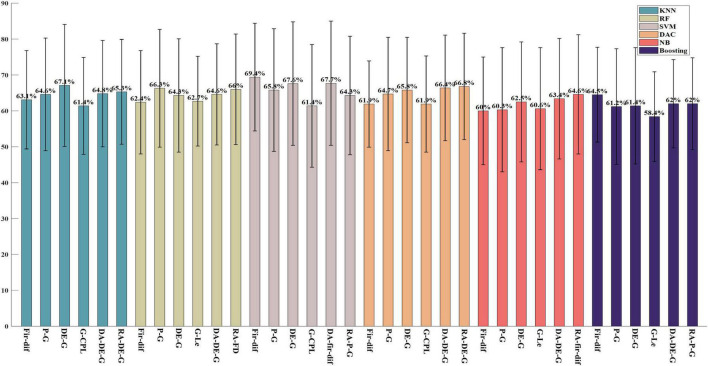
The highest classification accuracy for high and low valence emotion in each feature domain on the DEAP. The block which including six bars of same color is the results of same classifier and from left to right, it is the classification results by classifiers KNN, RF, SVM, DAC, NB, and Boosting, respectively.

As shown in Figure 3, on the DEAP dataset, the classification accuracy of various features for high and low valence emotions is not less than 50%, and the first-order differential features achieved the highest classification accuracy of 69.4% under the SVM classifier. Among the time-domain features, the first-order differential features achieved the highest classification accuracy of 69.4% under the SVM classifier. In frequency-domain features, the classification performance of gamma-band power features is the best, and the classification accuracy is 66.3% under the RF classifier. In the time-frequency domain, the gamma band differential entropy obtained the highest classification accuracy of 67.6% under the SVM classifier. Among the brain network properties, the gamma band local efficiency extracted from the coherence network achieved the highest classification accuracy of 62.7% under the RF classifier. Among the DA features, the gamma band DE obtained the highest classification accuracy of 66.4% under the DAC classifier. The RA features extracted from gamma band DE features performed the best and achieved the highest classification accuracy of 66.8% under the DAC classifier. On the DEAP dataset, it was also found that the features in higher frequency bands performed better than those in lower frequency bands, and the gamma band features achieved the highest classification accuracy, which is consistent with the results on the ESD dataset. Comparing the performance of different classifiers, it can be found that SVM and DAC classifiers have better classification performance.

**FIGURE 4:**
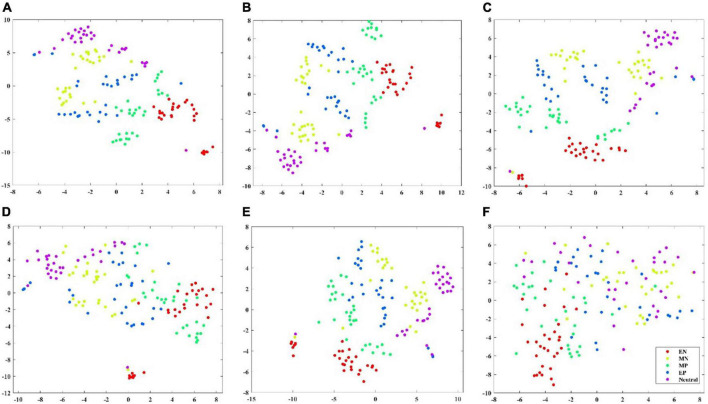
Feature visualization map on ESD dataset. **(A)** Second-order difference; **(B)** first-order difference; **(C)** fractal dimension; **(D)** high gamma band power; **(E)** high gamma band differential entropy; **(F)** high gamma band CPL of coherence network.

**FIGURE 5:**
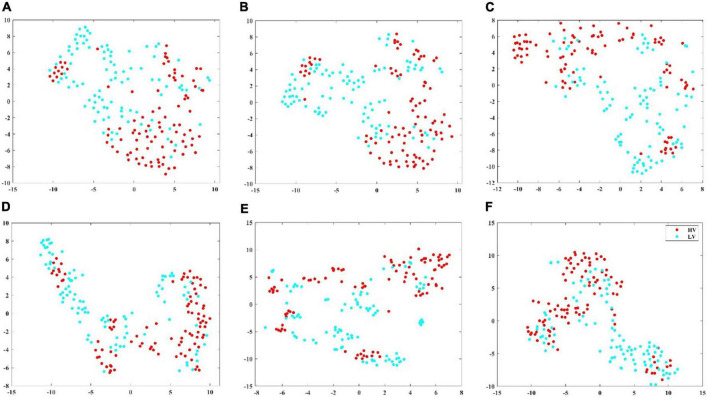
Feature visualization map on DEAP dataset. **(A)** Second-order difference; **(B)** first-order difference; **(C)** fractal dimension; **(D)** gamma band power; **(E)** gamma band differential entropy; **(F)** gamma band CPL of coherence network.

To show the classification performance of different features visually, this study selected one subject’s data and adopted the t-SNE algorithm to map high-dimensional features to two-dimensional space and compare the distribution of features within and between classes. On the ESD dataset, six types of features were selected, namely, first-order difference, second-order difference, FD, high gamma power, high gamma differential entropy, and high gamma band CPL of the coherence network, to visualize by t-SNE. On the DEAP dataset, first-order difference, second-order difference, FD, gamma-band power, gamma band DE, and gamma band Le of the coherence network were selected to visualize by t-SNE (Donahue et al., 2013). Figures 4, 5 show the feature distribution maps of the ESD and DEAP dataset. The feature distribution map shown in Figure 4 indicates that the second-order difference, FD, high gamma power, and high gamma DE, which obtained higher classification accuracy on the ESD dataset have a small distance within the class and a large distance between classes. From Figure 5, it can be seen that the first-order difference, second-order difference, and gamma DE on the DEAP dataset have a small distance within the class and a large distance between classes. The feature visualization results explain why the features performed better in the classification of different valence emotions, i.e., features with a small intra-class distance and a large inter-class distance can achieve higher accuracy.

### Important electrodes

From the classification results on both the ESD dataset and the DEAP dataset, it can be found that the features with better classification performance are the first-order difference, the second-order difference in the time domain, the high-frequency band power, and the high-frequency band DE features. Based on these four features, this study adopted the mRMR algorithm to reduce the feature dimension and find the most important electrodes on the ESD dataset and the DEAP dataset, respectively. On the ESD dataset, the most important 1-dimension, 4-dimension, 8-dimension, and 16-dimension features were selected from all 62 dimensions of each feature, and on the DEAP dataset, the features were selected from all 32 dimensions of each feature.

**FIGURE 6:**
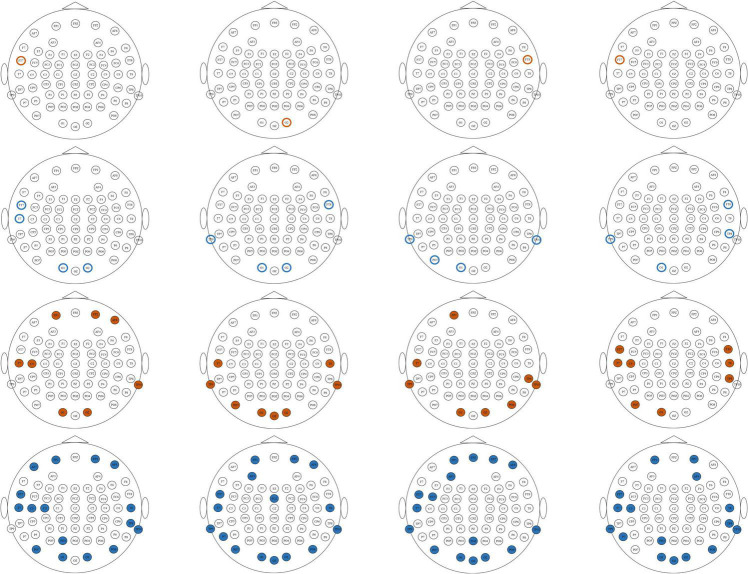
The distribution of the most important1, 4, 8, and 16 electrodes on the ESD dataset. Brown circles mark the most important 1 electrode, blue circles mark the most important 4 electrodes, brown solid circles mark the most important 8 electrodes, and blue solid circles mark the most important 16 electrodes. From top to bottom, each row corresponds to first-order difference, second-order difference, high gamma band power, and high gamma band DE, respectively.

**FIGURE 7:**
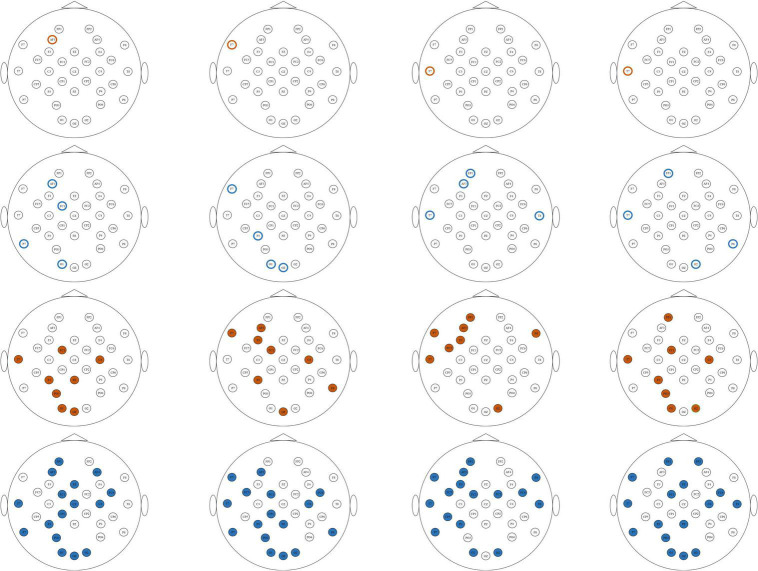
The distribution of the most important 1, 4, 8, and 16 electrodes on the DEAP dataset. Brown circles mark the most important 1 electrode, blue circles mark the most important 4 electrodes, brown solid circles mark the most important 8 electrodes, and blue solid circles mark the most important 16 electrodes. From top to bottom, each row corresponds to first-order difference, second-order difference, high gamma band power, and high gamma band DE, respectively.

Figures 6, 7 show the most important 1, 4, 8, and 16 electrodes of the first-order difference, second-order difference, high-frequency band power, and high-frequency band DE on the ESD dataset and the DEAP dataset, respectively. The blue rotundities in the figure represent the selected electrodes. From the results in the table, it can be seen that the electrodes from the prefrontal and temporal lobes are important for selecting the most important 1-dimension and 4-dimension features, and the features from the electrodes distributed in the occipital lobe are also selected when choosing more features.

**FIGURE 8:**
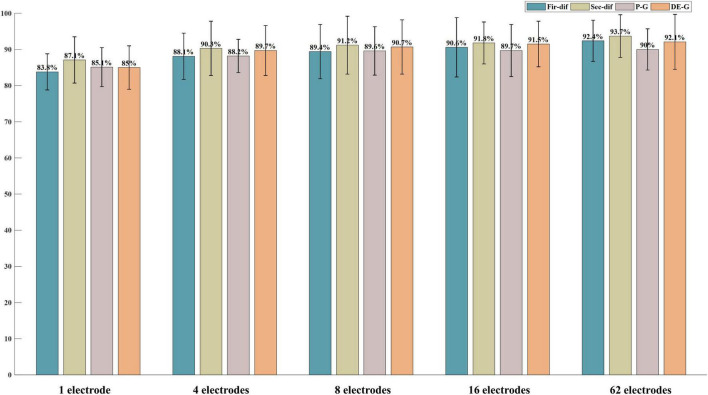
The classification results of features from 1, 4, 8, 16, and all 62 electrodes on the ESD dataset.

**FIGURE 9:**
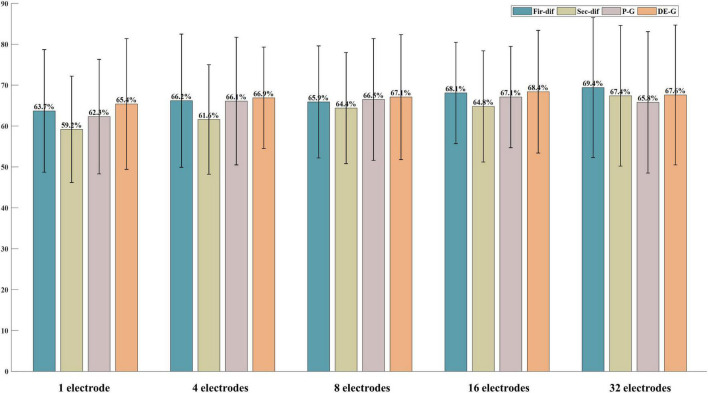
The classification results of features from 1, 4, 8, 16, and all 32 electrodes on the DEAP dataset.

According to the above classification performance comparison results, the first-order difference, second-order difference, high-frequency band energy, and high-frequency band DE show better classification performance. Then, this study investigated the classification performance of the four features extracted from the above-mentioned most important 1, 4, 8, 16 electrodes and all electrodes. Figures 8, 9 show the classification results of the features extracted from different numbers of electrodes on the ESD dataset and DEAP dataset, respectively. It can be seen from the results that the more electrodes are used, the higher the classification accuracy is. On the ESD dataset, when the number of electrodes is reduced by 3/4, i.e., using 16 electrodes located in the frontal lobe, temporal lobe, and occipital lobe, the classification accuracy is only 2% lower than that using all 62 electrodes. On the DEAP dataset, when only 1/2 of all electrodes are used, the gamma band DE even achieved 0.8% higher accuracy than that of using all 32 electrodes, and the accuracies of the first-order difference, second-order difference, and gamma band power decreased.

### Computing time

In practical applications, the feature extraction time should be as short as possible. In this paper, the computing time of features in different feature domains was compared. The computer used in this experiment is the AMAX server equipped with two Intel (R) Xeon (R) Gold 5120 2.20GHz CPUs, 256 GB RAM, and two NVIDIA Titan RTX GPUs, and running the 64-bit windows10 operating system. The data processing of this study was conducted on MATLAB 2018a. The feature extraction time was compared based on one sample 62*1200 (62 indicates the electrode number, 1200 indicates the data length, and the sampling rate is 600 Hz) in the ESD dataset, and the MATLAB commands “tic” and “toc” were used to record the computing time of different features. The calculation times of each feature in different feature domains are presented in Table 2.

**TABLE 2:** Calculation time of different features (s).

Time domain	ApEn	SamEn	FD	Fir-dif	Sec-dif	N-fir-dif	N-sec-dif	Std
	
	3.053	6.650	0.029	0.003	0.004	0.006	0.006	0.005
Frequency/time-frequency	PSD	Power	DE					
	0.602	0.208	0.118					
PLV	CC	CPL	Le	Ge				
	0.464	0.442	2.926	0.435				
Pearson	CC	CPL	Le	Ge				
	0.027	0.058	2.577	0.043				
Coherence	CC	CPL	Le	Ge				
	54.338	58.726	56.025	52.621				

It can be seen from Table 2 that the features whose calculation time is less than 0.1 s are FD, first-order difference, second-order difference, normalized first-order difference, normalized second-order difference, standard deviation, CC, CPL, and Ge extracted from the Pearson correlation network. The brain network features extracted from the coherence network need a long computing time, and the computing time is more than 50 s.

## Discussion

### Classification performance of features

Through the classification results on the ESD dataset and DEAP dataset, it can be found that the four features, namely the first-order difference, second-order difference, high-frequency band power, and high-frequency band DE performed better for the classification of different valence emotions. Specifically, among the four features, the classification performance of time-domain first-order difference and second-order difference under the SVM classifier achieved higher accuracy than that of the most used band power and differential entropy. Lan et al. (2016) also reported that the time-domain features have better classification performance than frequency features. Meanwhile, the feature visualization results show that the first-order difference and second-order difference have a large inter-class distance and a small intra-class distance while characterizing different valence emotions.

From the above results, the feature that performed the best in each feature domain while classifying different valence emotions can be found. Among time-domain features, the first-order difference and second-order difference achieved higher accuracy than other features. In the frequency domain, the high-frequency band power performed better than that in low-frequency bands on both ESD and DEAP datasets. Moreover, on the ESD dataset, the high gamma band power achieved the highest accuracy among all frequency-domain features, and on the DEAP dataset, the gamma band power obtained the highest accuracy. For the time-frequency domain, high-frequency band DE features perform better than those of the low-frequency band, and the high gamma band and gamma band DE achieved the highest accuracy on the ESD and DEAP dataset, respectively. Both frequency domain and time-frequency domain features show that a higher frequency band feature can achieve higher classification accuracy in the classification of different valence emotions. Many previous studies have also reported that high-frequency EEG features have a better performance in emotion recognition (Zheng and Lu, 2015; Zhuang et al., 2018). Our previous studies also proved the effectiveness of high-frequency features in emotion recognition (Yang et al., 2020). This study also explored the performance of brain network features that are widely used in recent years’ research. Three brain network calculation methods were used in this study, and then four network attributes were extracted as features. The results showed that the network feature calculated based on the Pearson correlation network had better performance, and the classification performance of CPL shows that it is more effective in characterizing different valence emotions. For DA and RA features, it can be found that the performance of RA features is slightly better than that of DA features. In addition, the performance of spatial asymmetric features is related to the original feature, i.e., if the classification performance of the original feature is good, the classification performance of the corresponding asymmetric feature is also good. This may be because both RA and DA features are both simple linear transformations of the original features. Through the classification results of features in different domains, it can be found that when classifying different valence emotions, the commonly used frequency domain and time-frequency domain features characterizing the rhythm features of EEG should be considered, and more attention should be given to the time-domain features that representing the time-varying information of EEG signals. Overall, when classifying different valence emotions with a first-order difference, second-order difference, high-frequency band power, and high-frequency band DE can achieve better classification results than other features.

### Important electrodes

Figures 6, 7 show the most selected electrodes and their distributions in the classification of different valence emotions. The most important features are extracted from similar electrodes of different features, and the results on ESD and DEAP datasets are consistent. The features are mainly extracted from the electrodes distributed at the frontal lobe, occipital lobe, and temporal lobe. Meanwhile, according to previous research, the frontal lobe is the brain region that executes high-level cognitive functions, including emotion processing and memory, and the occipital lobe mainly processes visual information related to emotions. Zheng et al. (2019) also pointed out that the key brain regions for emotion recognition included the frontal lobe, temporal lobe, and occipital lobe. Shuang et al. (2018) also found that the electrodes of the frontal lobe, temporal lobe, occipital lobe, and other brain regions are more important in exploring key electrodes for emotion classification (Liu et al., 2018). According to the results in this paper and previous studies, it can be found that different valence emotions have stable EEG patterns, and the prefrontal lobe, occipital lobe, and temporal lobe play an important role in characterizing different valence emotions. Additionally, the classification performance of different dimensions features was compared in this study, and it was found that the classification accuracy decreases with the reduction in the number of feature dimensions. On the ESD dataset, when the number of electrodes is reduced by 3/4, the classification accuracy is only about 2% lower than that of using all 62 electrodes. On the DEAP dataset, the accuracy of extracting the gamma band DE from only half of all electrodes is higher than that of using all electrodes. These classification results indicate that it is feasible to recognize different valence emotions based on a few electrodes, which can reduce computing complexity and is more convenient in actual applications. Therefore, the designing of an EEG acquisition device with a few electrodes or classifying different valence emotions based on a few electrodes can refer to the electrodes located in the frontal lobe, temporal lobe, and occipital lobe.

### Calculation time of features

Feature computing time is also very important for emotion recognition, especially in online emotion recognition because it affects the result feedback of emotion recognition. This study compared the calculation time of different features and presented the features with less calculation time, which can be used as a reference for other studies. Meanwhile, it was found that most time-domain features can be extracted in a short time. Especially, the first-order difference and second-order difference features have low computational complexity, and they are suitable for real-time emotion recognition situations.

### Comparison of classifiers

In this study, six commonly used shallow classifiers are used. As shown in the Figures 1–3, in each figure every block which including six bars of same color is the classification results of each classifier, and the same sequence location of each block is the result of same feature domain. By comparing the results of the same sequence location of each block in same figure, it can be seen that when feature is fixed, classifiers has different influence on classification results. The comparison results show that the SVM classifier has better performance in two-class classification tasks on both the ESD dataset and DEAP dataset, and the highest classification accuracy of different feature domains is mostly achieved by the SVM classifier. In the classification of five valence emotions, both SVM and DAC classifiers can obtain excellent results, and for some features, the DAC classifier may obtain better results than the SVM classifier. By comparing each block including six bars of same color, it can be seen, when classifier is fixed, the classification is decided by feature. And among all the features, the first-order difference, the second-order difference, the high-frequency band power and the high-frequency band differential entropy performed better. Generally, if we want to achieve the highest classification accuracy, we not only need to select feature but also need to select classifiers, the optimal combination of classifier and feature is required.

### Limitations

The limitation of this study is that it only explored the features for different valence emotions, but the effective features for different arousal emotions were ignored. Meanwhile, the combination of different features can provide complementary information and may contribute to better classification performance, so feature fusion methods will be explored in our future work.

## Conclusion

This manuscript systematically evaluated the performance of 110 features extracted from the time domain, frequency domain, time-frequency domain, spatial domain, and brain network on our self-built ESD dataset of 40 subjects and the public dataset DEAP. Meanwhile, the classification performance, computing time, and important electrodes of each feature were systematically analyzed and compared. From the experimental results, it can be seen that the first-order difference, second-order difference, high-frequency power, and high-frequency DE features outperform other features for the recognition of different valence emotions. Also, most time-domain features have less computing time than other features, which are more suitable for online emotion recognition. Besides, the electrodes in the frontal lobe, temporal lobe, and occipital lobe are more important for the recognition of different valence emotions, and when the number of electrodes is reduced by 3/4, the classification accuracy of features from 16 electrodes located in these brain regions is 91.8%, which is only about 2% lower than that of using all electrodes. In addition, the SVM classifier outperforms other shallow classifiers used in this study, and most features can obtain the highest accuracy with SVM. In the future, we will explore effective feature fusion methods in emotion recognition.

## Data availability statement

The raw data supporting the conclusions of this article will be made available by the authors, without undue reservation.

## Author contributions

KY was mainly responsible for the research design, data collection, data analysis, and manuscript writing of this study. LT was mainly responsible for research design and data analysis. YZ was mainly responsible for experiment design, data analysis, and manuscript writing. RZ was mainly responsible for data analysis and classification algorithm. RL was mainly responsible for production of figures and document retrieval. YG was mainly responsible for manuscript writing and document retrieval. BY was mainly responsible for research design and manuscript writing. All authors contributed to the article and approved the submitted version.

## References

[B1] Alkan A. Günay M. (2012). Identification of EMG signals using discriminant analysis and SVM classifier. *Expert Syst. Appl.* 39 44–47. 10.1016/j.eswa.2011.06.043

[B2] An Y. Hu S. Duan X. Zhao L. Xie C. Zhao Y. (2021). Electroencephalogram emotion recognition based on 3D feature fusion and convolutional autoencoder. *Front. Comput. Neurosci.* 15:743426. 10.3389/fncom.2021.743426 34733148 PMC8558247

[B3] Bai L. Ma H. Huang Y. X. Luo Y. J. (2005). The development of native Chinese affective picture system-A pretest in 46 college students. *Chin. Ment. Health J.* 19 719–722.

[B4] Bhadra T. Bandyopadhyay S. (2021). Supervised feature selection using integration of densest subgraph finding with floating forward-backward search. *Inf. Sci.* 566 1–18. 10.1016/j.ins.2021.02.034

[B5] Blair K. S. Smith B. W. Mitchell D. Morton J. Vythilingam M. Pessoa L. (2007). Modulation of emotion by cognition and cognition by emotion. *NeuroImage* 35 430–440. 10.1016/j.neuroimage.2006.11.048 17239620 PMC1862681

[B6] Carretié L. Iglesias J. GarcA T. (1997). A study on the emotional-processing of visual stimuli through event-related potentials. *Brain Cogn.* 34 207–217. 10.1006/brcg.1997.0895 9220086

[B7] Chen C. Wang Q. H. Liu X. H. Liu F. (2013). The study of relationship between negative emotion and cognitive executive function. *Prog. Mod. Biomed.* 13 1149–1153.

[B8] Chen J. Wang L. Jia X. Zhang P. (2019). “EEG-based emotion recognition using deep convolutional neural network,” in *Proceedings of the 2019 IEEE 8th Data Driven Control and Learning Systems Conference (DDCLS)* (Dali: IEEE).

[B9] Chen T. Ju S. Ren F. Fan M. Gu Y. (2020). EEG emotion recognition model based on the LIBSVM classifier. *Measurement* 164 1–13. 10.1016/j.measurement.2020.108047

[B10] Ding C. Peng H. (2005). Minimum redundancy feature selection from microarray gene expression data. *J. Bioinformatics Comput. Biol.* 3 185–205. 10.1142/S0219720005001004 15852500

[B11] Donahue J. Jia Y. Vinyals O. Hoffman J. Darrell T. (2013). “DeCAF: A deep convolutional activation feature for generic visual recognition,” in *Proceedings of the 31st international conference on machine learning.* (Tianjin).

[B12] Duan R. N. Wang X. W. Lu B. L. (2012). “EEG-based emotion recognition in listening music by using support vector machine and linear dynamic system,” in *Proceedings of the 19th international conference on neural information processing* (Berlin: Springer), 468–475. 10.1007/978-3-642-34478-7_57

[B13] Gonuguntla V. Veluvolu K. C. Kim J. H. (2020). *Recognition of event-associated brain functional networks in EEG for brain network based applications.* Iowa, IA: IEEE, 271–274. 10.1109/ISBI45749.2020.9098708

[B14] He T. Zhang Z. Zhang H. Zhang Z. Li M. (2019). “Bag of tricks for image classification with convolutional neural networks,” in *Proceedings of the 2019 IEEE/CVF conference on computer vision and pattern recognition (CVPR)* (Long Beach, CA). 10.1109/CVPR.2019.00065

[B15] Hu X. Chen J. Wang F. Zhang D. (2019). Ten challenges for EEG-based affective computing. *Brain Sci. Adv.* 5 1–20. 10.26599/BSA.2019.9050005

[B16] Huang G. B. Zhu Q. Y. Siew C. K. (2006). Extreme learning machine: Theory and applications. *Neurocomputing* 70 489–501. 10.1016/j.neucom.2005.12.126

[B17] HyvRinen A. (1999). The fixed-point algorithm and maximum likelihood estimation for independent component analysis. *Neural Process. Lett.* 10 1–5. 10.1023/A:1018647011077

[B18] Jackson-Koku G. (2016). Beck depression inventory. *Occup. Med.* 18 174–175. 10.1093/occmed/kqv087 26892598

[B19] Jenke R. Peer A. Buss M. (2014). Feature extraction and selection for emotion recognition from EEG. *IEEE Trans. Affect. Comput.* 5 327–339. 10.1109/TAFFC.2014.2339834

[B20] Jia J. Yang N. Zhang C. Yue A. Yang J. Zhu D. (2013). Object-oriented feature selection of high spatial resolution images using an improved relief algorithm. *Math. Comput. Model.* 58 619–626. 10.1016/j.mcm.2011.10.045

[B21] Johnson R. (1995). On the neural generators of the P300: Evidence from temporal lobectomy patients. *Electroencephalogr. Clin. Neurophysiol.* 44 110–129. 7649013

[B22] Keller J. M. Gray M. R. Givens J. A. (2012). A fuzzy K-nearest neighbor algorithm. *IEEE Trans. Syst. Man Cybernet.* 15 580–585. 10.1109/TSMC.1985.6313426

[B23] Khare S. K. Bajaj V. (2021). Time-frequency representation and convolutional neural network-based emotion recognition. *IEEE Trans. Neural Netw. Learn. Syst.* 32 2901–2909. 10.1109/TNNLS.2020.3008938 32735536

[B24] Koelstra S. (2012). DEAP: A database for emotion analysis; using physiological signals. *IEEE Trans. Affect. Comput.* 3 18–31. 10.1109/T-AFFC.2011.15

[B25] Lan Z. Sourina O. Wang L. Liu Y. (2016). Real-time EEG-based emotion monitoring using stable features. *Vis. Comput.* 32 347–358. 10.1007/s00371-015-1183-y

[B26] Lang P. J. Bradley M. M. Cuthbert B. N. (2001). *International affective picture system (IAPS); instruction manual and effective ratings.* Technical Report A-4. Gainesville, FL: The Center for Research in Psychophysiology, University of Florida.

[B27] Li F. Bei C. He L. Tao Z. Fei W. Yi J. (2016). The time-varying networks in P300: A task-evoked EEG study. *IEEE Trans. Neural Syst. Rehabil. Eng.* 24 725–733. 10.1109/TNSRE.2016.2523678 26849870

[B28] Li M. Xu H. Liu X. (2018). Emotion recognition from multichannel EEG signals using K-nearest neighbor classification. *Technol. Health Care* 26 509–519. 10.3233/THC-174836 29758974 PMC6027901

[B29] Li P. Y. Liu H. Si Y. Li C. Li F. Zhu X. (2019). EEG based emotion recognition by combining functional connectivity network and local activations. *IEEE Trans. Biomed. Eng.* 66 2869–2881. 10.1109/TBME.2019.2897651 30735981

[B30] Li X. Li X. Luo Y. J. Luo Y. (2006). Differential influences of negative emotion on spatial and verbal working memory: Evidence from event-related potential and source current density analysis. *Neuroreport* 17 1555–1559. 10.1097/01.wnr.0000234744.50442.2b 16957607

[B31] Liaw A. Wiener M. (2002). Classification and regression by randomForest. *R News* 2 18–22.

[B32] Liu S. Chen L. Guo D. Liu X. Sheng Y. Ke Y. (2018). Incorporation of multiple-days information to improve the generalization of eeg-based emotion recognition over time. *Front. Hum. Neurosci.* 12, 267–276. 10.3389/fnhum.2018.00267 30013470 PMC6036248

[B33] Lukosevicius M. Jaeger H. (2009). Reservoir computing approaches to recurrent neural network training. *Comput. Sci. Rev.* 3 127–149. 10.1016/j.cosrev.2009.03.005

[B34] Meng X. Yuan J. Hong L. (2009). Automatic processing of valence differences in emotionally negative stimuli: Evidence from an ERP study. *Neurosci. Lett.* 464 228–232. 10.1016/j.neulet.2009.08.064 19720111

[B35] Morris J. D. (1995). Observations: SAM: The selfAssessment Manikin; an efficient cross-cultural measurement of emotional response. *J. Adv. Res.* 35 63–68.

[B36] Nie D. Wang X. W. Shi L. C. Lu B. L. (2011). “EEG-based emotion recognition during watching movies,” in *Proceedings of the 2011 5th international IEEE/EMBS conference on neural engineering (NER).* (Cancun), 667–670. 10.1109/NER.2011.5910636

[B37] Peng Z. Wei S. Tian J. Qi Z. Bo X. (2016). “Attention-based bidirectional long short-term memory networks for relation classification,” in *Proceedings of the 54th annual meeting of the association for computational linguistics*, Vol. 2. (Berlin: Association for Computational Linguistics).

[B38] Qian Y. Tan J. Chen A. (2015). The stages of information processing and emotional stimuli processing. *J. Psychol. Sci.* 38 801–806.

[B39] Rish I. (2001). An empirical study of the naive bayes classifier. *J. Univers. Comput. Sci.* 1:127.

[B40] Saeedreza E. Rezazadeh S. A. Moussavi S. Z. Ali S. (2009). A new pattern recognition technique in non destructive testing by the use of linear discriminate analysis. *Mod. Appl. Sci.* 3 118–126. 10.5539/mas.v3n5p118

[B41] Saha P. Fels S. (2019). Hierarchical deep feature learning for decoding imagined speech from EEG. *Proc. AAAI Conf. Artif. Intell.* 33, 10019–10020. 10.1609/aaai.v33i01.330110019

[B42] Santamaria-Vazquez E. Martinez-Cagigal V. Vaquerizo-Villar F. Hornero R. (2020). EEG-Inception: A novel deep convolutional neural network for assistive ERP-based brain-computer interfaces. *IEEE Trans. Neural Syst. Rehabil. Eng.* 28 2773–2782. 10.1109/TNSRE.2020.3048106 33378260

[B56] Shuang L. Chen L. Guo D. Liu X. Yue S. (2018). Incorporation of multiple-days information to improve the generalization of EEG-based emotion recognition over time. *Front. Hum. Neurosci.* 12:267. 10.3389/fnhum.2018.00267 30013470 PMC6036248

[B43] Sun Y. Kamel M. S. Wong A. Yang W. (2007). Cost-sensitive boosting for classification of imbalanced data. *Pattern Recognit.* 40 3358–3378. 10.1016/j.patcog.2007.04.009

[B44] Van Straaten E. C. W. Stam C. J. (2013). Structure out of chaos: Functional brain network analysis with EEG, MEG, and functional MRI. *Eur. Neuropsychopharmacol.* 23 7–18. 10.1016/j.euroneuro.2012.10.010 23158686

[B45] Wang H. Liu L. (2020). Experimental investigation about effect of emotion state on people’s thermal comfort. *Energy Build.* 211:109789. 10.1016/j.enbuild.2020.109789

[B46] Wang J. Wang M. (2021). Review of the emotional feature extraction and classification using EEG signals. *Cogn. Rob.* 1 29–40. 10.1016/j.cogr.2021.04.001

[B47] Wilson K. A. Chambless D. L. Beurs E. D. (1999). “Beck anxiety inventory,” in *The use of psychological testing for treatment planning and outcomes assessment*, ed. Maruish M. E. (Hillsdale, NJ: Lawrence Erlbaum Associates Publishers), 971–992.

[B48] Wu X. Zheng W. L. Li Z. Lu B. L. (2022). Investigating EEG-based functional connectivity patterns for multimodal emotion recognition. *J. Neural Eng.* 19:016012. 10.1088/1741-2552/ac49a7 35094982

[B49] Yang K. Tong L. Shu J. Zhuang N. Zeng Y. (2020). High gamma band EEG closely related to emotion: Evidence from functional network. *Front. Hum. Neurosci.* 14:89. 10.3389/fnhum.2020.00089 32265674 PMC7107011

[B50] Yuan J. Zhang Q. Chen A. Li H. Wang Q. Zhuang Z. (2007). Are we sensitive to valence differences in emotionally negative stimuli? Electrophysiological evidence from an ERP study. *Neuropsychologia* 45 2764–2771. 10.1016/j.neuropsychologia.2007.04.018 17548095

[B51] Zheng W. L. Lu B. L. (2015). Investigating critical frequency bands and channels for EEG-based emotion recognition with deep neural networks. *IEEE Trans. Auton. Ment. Dev.* 7 162–175. 10.1109/TAMD.2015.2431497

[B52] Zheng W.-L. Liu W. Lu Y. Cichocki A. Lv B.-L. (2019). EmotionMeter: A multimodal framework for recognizing human emotions. *IEEE Trans. Cybern.* 49 1110–1122. 10.1109/TCYB.2018.2797176 29994384

[B53] Zhong P. Wang D. Miao C. (2020). EEG-based emotion recognition using regularized graph neural networks. *IEEE Trans. Affect. Comput.* 1 1–12. 10.1109/TAFFC.2020.2994159

[B54] Zhou B. Khosla A. Lapedriza A. Oliva A. Torralba A. (2016). “Learning deep features for discriminative localization,” in *Proceedings of the 2016 IEEE conference on computer vision and pattern recognition CVPR* (Las Vegas, NV: IEEE). 10.1109/CVPR.2016.319

[B55] Zhuang N. Ying Z. Kai Y. Chi Z. Tong L. Yan B. (2018). Investigating patterns for self-induced emotion recognition from EEG signals. *Sensors* 18 841–862. 10.3390/s18030841 29534515 PMC5877378

